# Multiple diversity concepts and their ethical-epistemic implications

**DOI:** 10.1007/s13194-018-0209-5

**Published:** 2018-06-15

**Authors:** Daniel Steel, Sina Fazelpour, Kinley Gillette, Bianca Crewe, Michael Burgess

**Affiliations:** 1 W. Maurice Young Centre for Applied Ethics, University of British Columbia, 6356 Agricultural Road, Vancouver, BC V62 1Z2 Canada; 2 Department of Philosophy, University of British Columbia, 1866 Main Mall, Vancouver, BC V6T 1Z1 Canada

**Keywords:** Diversity, Social epistemology, Deliberative mini-publics

## Abstract

A concept of diversity is an understanding of what makes a group diverse that may be applicable in a variety of contexts. We distinguish three diversity concepts, show that each can be found in discussions of diversity in science, and explain how they tend to be associated with distinct epistemic and ethical rationales. Yet philosophical literature on diversity among scientists has given little attention to distinct concepts of diversity. This is significant because the unappreciated existence of multiple diversity concepts can generate unclarity about the meaning of “diversity,” lead to problematic inferences from empirical research, and obscure complex ethical-epistemic questions about how to define diversity in specific cases. We illustrate some ethical-epistemic implications of our proposal by reference to an example of deliberative mini-publics on human tissue biobanking.

## Introduction

Philosophers and social scientists have explored ways in which diversity might lead to epistemically better science or improve group performance in a variety of settings (Aggarwal and Wooley 2013; Bear and Wooley 2011; Engel et al. 2014; Harding 2015; Hong and Page 2004; Intemann 2010a; Kitcher 1990; Levine et al. 2014; Longino 1990, 2002; Lount and Phillips 2007; Loyd et al. 2013; Muldoon 2013; Page 2017; Page 2007; Phillips 2014; Phillips et al. 2009; Solomon 2001; Zollman 2010; Woolley et al. 2010). In these literatures, diversity may pertain to social position, identity, value perspectives, theoretical orientation, task-related skills, and so on. For convenience, let the term *context of diversity* refer to a background situation that suggests attributes relevant for assessing diversity.^1^ Distinct attributes are likely to be judged relevant depending on whether the diversity of an incoming class of students, a scientific research team, or a biotechnology firm is in question. In this article, our focus is not on contexts of diversity, but instead on the shared meaning of “diversity” across contexts.

We use the term *concept of diversity* to refer to an understanding of what constitutes diversity, abstracted from questions of which attributes are relevant to diversity in a specific context. While the existence of multiple contexts of diversity is widely recognized, philosophical discussions of how diversity might enhance science have devoted little attention to concepts of diversity in our sense. This situation is problematic for several reasons. First, several diversity concepts exist, and there are substantial differences among them. We distinguish two general types of diversity concept, *within group* and *comparative*, and focus on three specific diversity concepts that fall under these two headings, *egalitarian*, *representative*, and *normic*. To our knowledge, no previous work has explicitly distinguished all three of these concepts or explored their interconnections. Nevertheless, each can be identified in work on diversity in science or in explanations of its cognitive benefits. Thus, failure to attend to these concepts and their differences can result in a general lack of clarity about the intended meaning of the word “diversity” as well as problematic inferences from empirical evidence to conclusions about diversity’s effects.

In addition, inattention to diversity concepts tends to obscure important coupled ethical-epistemic issues. One type of ethical-epistemic coupling occurs when “value decisions embedded in research models and methods … go unquestioned and often unappreciated” (Tuana 2010, p. 1957; cf. Fehr and Plaisance 2010). We suggest that such a phenomenon is present in explanations, proposed by philosophers and social scientists of various stripes, of how diversity can enhance the cognitive performance of groups. These explanations sometimes presuppose distinct conceptions of diversity that are associated with differing ethical and epistemic reasons for why diversity should be valued. We explore some implications this idea with an example of deliberative mini-publics used to inform science policy issues, wherein we suggest that ethical-epistemic concerns can result in the use of hybrid (e.g., representative/normic) diversity concepts.

The remainder of this paper is organized as follows. In section 2, we review literature on diversity concepts. Section 3 elaborates egalitarian, representative, and normic diversity concepts, while section 4 demonstrates their relevance to discussions of diversity in science or to explanations of the effects of diversity on group performance. Section 5 discusses deliberative mini-publics on biobanking to illustrate ethical-epistemic issues related to multiple diversity concepts.

## Previous work on diversity concepts

A concept of diversity, as we use the term, is an understanding of what makes a group diverse that may be applicable in a variety of contexts. Specifically, concepts of diversity need not be tied to an attribute, such as gender, theoretical perspective, and so forth. Thus, we do not review distinctions drawn among attributes, such as surface versus deep, demographic versus cognitive, task-related versus non-task-related, or functional versus identity (Harrison and Sin 2006: 2; Harrison and Klein 2007: 1199; Hong and Page 2004). In addition, we distinguish concepts from measures of diversity, which we understand as mathematical formulas for quantifying the degree to which a group is diverse. Usually, the relationship between concepts and measures of diversity is one to many: for a given diversity concept, several measures may be reasonably taken to quantify it (McDonald and Dimmick 2003; Solanas et al. 2012; Teachman 1980). Since concepts, as opposed to contexts, of diversity have not been clearly distinguished in philosophical literature on diversity among scientists, we review other literatures where they have been, namely, social science and ecology.

Discussions of diversity concepts can be found in social science literature, usually in connection with measures of diversity (Harrison and Klein 2007; McDonald and Dimmick 2003; Solanas et al. 2012; Teachman 1980). Teachman (1980) provides an early and influential example of such work, defining “population diversity” as “the distribution of population elements (which are not limited to humans or the characteristics of humans) along a continuum of homogeneity to heterogeneity with respect to one or more variables” (Teachman 1980, 341). Three observations about this definition are helpful.

First, Teachman’s definition expresses a diversity concept in the sense described above. It explicitly abstracts from particular contexts and attributes, but it does not indicate a mathematical formula for quantifying degrees of diversity. Second, population diversity is a broad umbrella under which more specific diversity concepts might fall. The distinctive feature of these concepts is that, once the relevant variables or attributes are provided, the diversity of a population (or group) depends only on their distribution within that population (or group). Such diversity concepts differ only in terms of which distributions they take to exhibit greater or lesser heterogeneity. In this way, Teachman’s population diversity is very similar to our concept of *within group* diversity discussed in section 3. Third, the label “population diversity,” rather than simply “diversity,” suggests that other diversity concepts may exist. What we call *comparative* diversity concepts diverge from Teachman’s notion of population diversity insofar as they depend on a comparison with a reference population.

Consider a more specific diversity concept suggested by Teachman himself under the guise of “desirable properties of a measure of qualitative variation” (Teachman 1980, 342). The term “qualitative variation” refers to variation in qualitative attributes, such as gender or religious affiliation, rather than variation along a quantitative scale, as might be used for income or height. Let A = {*a*_1_, …, *a*_*n*_} represent a qualitative attribute, where A is a label for the attribute and *a*_1_, …, *a*_*n*_ are the mutually exclusive categories into which it subdivides. For example, if the attribute is religious affiliation, then the categories might be {Buddhism, Christianity, Hinduism, Islam, Judaism, Secularism, Sikhism}.^2^ While multiple attributes (e.g., gender, ethnicity, and religion) might be considered in a single case, as Teachman notes, for simplicity we limit attention here to examples involving only one. Finally, let G denote the focal group, or population, whose diversity is in question. Focal groups can be almost any collection of people (inhabitants of a town, students at a university, a committee, a board of directors, a legislature, a research team, an expert panel, etc.) or other entities (e.g., species in an ecosystem, segments of time devoted to distinct perspectives in media coverage of a topic, etc.).

Teachman’s “desirable properties” for a measure of diversity for qualitative variables, then, are as follows (Teachman 1980, 342):

1. Diversity is minimized when everyone (or everything) in G falls in a single category *a*_*i*_ ∈ A;
2. Diversity is maximized when each category in A is present in G at a proportion of 1/*n*, and
3. For any groups G and G’, if *i* categories of A are present in G at proportions of 1/*i* each, *j* categories of A are present in G’ at proportions of 1/*j* each, and *i* > *j*, then G is more diverse than G’.

In the above example above, diversity would be minimized if everyone in the group had the same religious affiliation, for instance, if all were Buddhists. Conversely, diversity would be maximized if all affiliations were present in equal proportions. The third property tells us something about intermediate cases. Consider a group composed of equal numbers of Buddhists, Christians, Hindus, and Muslims, and a second group composed of equal numbers of Jews, Muslims, and Sikhs. Then (3) tells us that the first group is more diverse than the second.

Properties (1) through (3) suggest that diversity entails a uniform distribution over the full set of attribute categories. The more flat and spread the distribution is, the closer it approximates a uniform distribution, and hence the greater the diversity. The more concentrated the distribution is on a single category, the further removed it is from a uniform distribution, and consequently the less the diversity. This diversity concept is labeled “variety” by Harrison and Klein (2007) and “dual concept”^3^ by McDonald and Dimmick (2003, 64). We call the diversity concept picked out by Teachman’s three desirable properties “egalitarian,” which we take to be a more descriptive label. Numerous measures of egalitarian diversity for qualitative attributes exist, of which Blau’s index is the most common (Harrison and Klein 2007; Joshi et al. 2011, 525; McDonald and Dimmick 2003; Solanas et al. 2012; Teachman 1980).^4^

Egalitarian diversity is not the only diversity concept that has been proposed. Harrison and Klein propose three concepts of diversity—separation, variety, and disparity—and suggest that different measures are appropriate for each (Harrison and Klein 2007, 1202–1203, 1210–1214). As noted, “variety” is Harrison and Klein’s name for egalitarian diversity. Harrison and Klein’s innovation is to suggest that an egalitarian diversity concept is appropriate only for cases involving qualitative attributes, and that distinct concepts—separation and disparity—are associated with quantitative variables.

Diversity as separation is difference among group members “along a single continuous [and lateral] attribute” (Harrison and Klein 2007, 1203). The key idea is that separation involves difference of degree, but without any clear difference of social status. Examples include the degree of group members’ “organizational commitment” and “perceptions of leader charisma” (Harrison and Klein 2007, 1203). In these examples, diversity depends on the extent of divergence among the viewpoints or attitudes present in the group. Consequently, diversity as separation is maximized by polarization, that is, when the group is divided into equal numbers at opposite extremes (Harrison and Klein 2007, 1204). Finally, diversity as disparity is difference along a single continuous attribute, albeit a “vertical” attribute that is both socially valued and socially consequential (Harrison and Klein 2007, 1206). Examples include “power” and “pay” (Harrison and Klein 2007, 1206). Intuitively, diversity as disparity is the extent of imbalance between the best off and everyone else. Consequently, it is maximized by a positively skewed distribution, that is, by a distribution in which one individual occupies the highest point of the scale and all others the lowest (Harrison and Klein 2007, 1203).

The literature reviewed thus far, then, specifies egalitarian diversity for qualitative attributes but suggests that there may be other diversity concepts for quantitative variables. However, it is problematic to use the distinction between qualitative and quantitative attributes as a basis for categorizing diversity concepts. First, whether qualitative or quantitative variables are used is a practical decision of representation and modeling, not underlying concepts. For example, income can be represented by a quantitative variable, but it can also be represented by a set of qualitative categories (e.g., low income, middle income, high income). Yet in either case, the undelying concept might be that the more flat and spread the distribution, the greater the diversity. Second, the distinction between qualitative categories and quantitative variables is not always as sharp as some diversity researchers appear to suggest. There is now a burgeoning literature offering various, quantitatively well-specified models of qualitative categories (Minda and Smith 2001; Danks 2014; Gopnik and Wellman 2012). These quantitative models can provide *similarity measures* for comparing different qualitative categories, which suggests that one could add a measure of similarity to an egalitarian concept. For example, when the attribute is religious affiliation, Christianity and Judaism might be judged more similar than Christianity and Sikhism, and consequently a group consisting of equal numbers of Christians and Jews might be considered less diverse than a group consisting of equal numbers of Christians and Sikhs. Degrees of similarity are closely linked to Harrison and Klein’s (2007) notion of diversity as separation. However, we think it is more fruitful to see similarity as a conceptual ingredient that might be incorporated in several diversity concepts, rather than as a stand-alone diversity concept tied to quantitative variables. In fact, one contributor to the literature on diversity concepts proposes that “the manner and degree” of similarity (or difference) is an essential dimension of diversity generally (Stirling 2007, 709; Stirling 1998, 39–40, 58).

Multiple diversity concepts are also found in debates about biodiversity. In ecology, a major question has been whether there is any place for concepts of diversity that are not tied in some way to the biological context (Pielou 1980; Sugihara 1982; Molinari 1989; Ricotta 2005). One aspect of this debate is about whether an egalitarian diversity concept is appropriate for the study of biodiversity (Junge 1994, 19). The alleged problem with conceiving of diversity in this way is that it separates ecological diversity from the very reason it is thought to be worth studying in the first place—namely, “its possible connection with the functioning and organization of communities” (Sugihara 1982, 564). In conservation biology, some also insist that concepts of diversity take into account the degree to which the elements (e.g., species) at a particular site are similar to each other, so that diversity is linked to the preservation of unique ecological components of a given community (Maclaurin and Sterelny 2008; Vane-Wright et al. 1991, 236–241).

Moreover, conservation biologists also often require a diversity concept that takes into account rarity relative to other sites (Margules et al. 1988; Vane-Wright et al. 1991, 241–245). Thus, conservation biologists, following Whittaker (1960), often distinguish between “α-diversity, the diversity within a site, β-diversity, that between sites, and γ-diversity, or the total diversity of a region, including both α- and β-diversity” (Sarkar 2006, 136). Sarkar argues that only the latter two concepts are relevant to biodiversity, and that the most common β-diversity concept is *complementarity* (Sarkar 2006, 137; Sarkar 2002, 153). If species are the relevant attribute, then an ecosystem at site S is complementary with respect to an ecosystem located in region R to the extent that it possesses species that are rare in R. Complementarity, then, is an example of what we call comparative diversity.

Some conservation biologists also argue that their field is, or should be, primarily concerned with the preservation of “native” species, “native” population structures of particular species, or some other “native” aspect, or aspects, of the ecosystem (see, e.g., Angermeier 1994). These conservation biologists are struck by the allegedly counterintuitive possibility of maintaining or even increasing the biodiversity of a community by introducing new species, modifying a species’ population structure, fragmenting a landscape, etc.—that is, actions that are supposedly at odds with the goals of the field (Angermeier 1994; Angermeier and Karr 1994, 694; Thompson and Starzomski 2007). One response to this situation is to adopt alternative diversity concepts that, as Angermeier and Karr put it, “incorporate explicit native criteria” (1994, 694).

In sum, this section has shown that (a) multiple diversity concepts exist, (b) some depend only on the distribution within the focal group, while others depend on a comparison with a reference population, (c) these concepts might or might not incorporate a similarity weighting, and (d) some depend on non-distributional considerations (e.g., “nativeness”). In what follows, we explain how all of these conceptual complexities also arise in discussions of diversity among scientists.

## Egalitarian, representative, and normic diversity

A concrete example will be helpful to convey the diversity concepts examined in this section. Consider the Vaisakhi 2017 celebration in Vancouver, Canada. For those unfamiliar with this holiday, Vaisakhi is a harvest and New Year’s festival originating from the Punjab region that is celebrated in the month of April by followers of the Sikh religion throughout the world. Vaisakhi 2017 in Vancouver was a major parade and street festival that attracted approximately 50,000 attendees, the majority of whom were Sikhs. The Vaisakhi parade in nearby Surrey, British Columbia drew an even larger crowd of around 300,000. Now consider this deceptively simple question: Was the collection of people attending these Vaisakhi celebrations diverse? Here are three possible answers:

1. Yes, because, unlike Canada as a whole, the majority were not Christians.
2. No, because the distribution was heavily skewed toward just one religious group rather than evenly spread across several religious persuasions.
3. Yes, because the major sects of Sikhism found in North America were proportionally represented.

The three answers above share a common background context, namely, the Vaisakhi 2017 festivals in metro-Vancouver, Canada—and they also take the religious affiliation to be the relevant attribute.^5^ Nevertheless, the answers and their rationales diverge from one another. That, we claim, is because they express three distinct conceptions of diversity, which we label egalitarian, representative, and normic.

As discussed in section 2, a group G is diverse in an egalitarian sense to the extent that the distribution in G over the relevant attribute A = {*a*_1_, …, *a*_*n*_} is uniform. This diversity concept is on display in answer 2 of the Vaisakhi example. Let the attribute be A = {Buddhism, Christianity, Hinduism, Islam, Judaism, Secularism, Sikhism}. Then from an egalitarian perspective, the diversity of the attendees of the Vaisakhi festival is close to the minimum, because almost all fall into a single category, namely, Sikhs.

Turn, then, to representative diversity. Let A be the relevant attribute and P the reference population; then G is diverse in a representative sense to the extent that the distribution of A in G is similar to that in P. Answer 3 in the Vaisakhi example expresses a representative concept of diversity. In that answer, the reference population consists of the North American Sikh community, and the attribute consists of distinct sects of Sikhism, for instance, A = {Orthodox, Niran Karis, Nam-Dharis, Akhand Kirtani Jatha, Sikh Dharma}. In answer 3, diversity is claimed on the grounds that the distribution of these sects among attendees of the Vaisakhi 2017 festivals in metro-Vancouver is similar to that of the reference population.

Consider the relationship between egalitarian and representative diversity concepts. When the attribute divides into just two categories, *a*_1_ and *a*_2_, egalitarian and representative diversity concepts coincide only when *a*_1_ are *a*_2_ are present in equal proportions in P. This situation is often assumed in discussions of gender diversity in science, technology, engineering, and mathematics (STEM) fields, which typically treat gender as split into two mutually exclusive and exhaustive categories present in roughly equal proportions in the general population (Hill et al. 2010; Xie and Shauman 2003). However, egalitarian and representative diversity concepts diverge in other cases. If the attribute is religious affiliation, as in the Vaisakhi example, then egalitarian diversity would be maximized when each affiliation is present in equal proportions, but representative diversity would not be maximized by this distribution for most reference populations.

In contrast, normic diversity concepts define diversity in relation to an assumed non-diverse attribute category^6^ in a reference population, which can reflect numerical majority or non-distributional factors such as social status, or both. According to a normic diversity concept, a group is diverse to the extent that its members diverge from the non-diverse norm. Normic diversity concepts, then, can be characterized as follows. Let the attribute be A = {*a*_1_, …, *a*_*n*_} and *a*_*nd*_ ∈ A be the non-diverse norm in the reference population P; then group G is diverse in a normic sense with respect to P to the extent that few individuals in G are *a*_*nd*_. Answer 1 of the Vaisakhi example suggests a normic diversity concept, with Christian as the non-diverse norm in the reference population.

Normic diversity possesses several striking features in comparison to egalitarian and representative concepts. One of these is that a group in which every person falls into the same category of the relevant attribute can be maximally diverse in a normic sense. Yet such a situation would constitute the theoretical minimum of egalitarian diversity, and would be non-diverse in a representative sense unless the reference population were similarly homogeneous. Moreover, a normic concept is compatible with declaring a single person diverse, which would be absurd from an egalitarian perspective. Nevertheless, normic diversity bears important similarities to the notion of diversity as complementarity discussed in connection with biodiversity. A group is diverse in a complementary sense when its distribution is the inverse of the reference population. The attendees at the Vancouver Vaisakhi festival might be considered diverse in this way, since a religious affiliation that is a minority in Canada as a whole predominates among the festival attendees.^7^

However, normic diversity differs from complementarity in that the non-diverse norm in a population is not necessarily determined by the distribution in the reference population. Consequently, in some cases the non-diverse norm might be present in lesser proportions than other categories of the relevant attribute. For example, consider a patriarchal society in which the proportion of women is slightly greater than that of men. If the relevant attribute were binary gender, then men could be taken to be the non-diverse norm and a group of women might be considered diverse in a normic sense. However, a group in which women were the majority would not be diverse in a complementary sense in this example, as complementarity would entail a slight majority of men. Normic diversity, therefore, can depend on non-distributional factors. In this way, social status is analogous to “nativeness” in discussions of biodiversity. Both reflect concerns apart from the distribution in the reference population that identify certain attribute categories as non-diverse.

**Fig 1:**
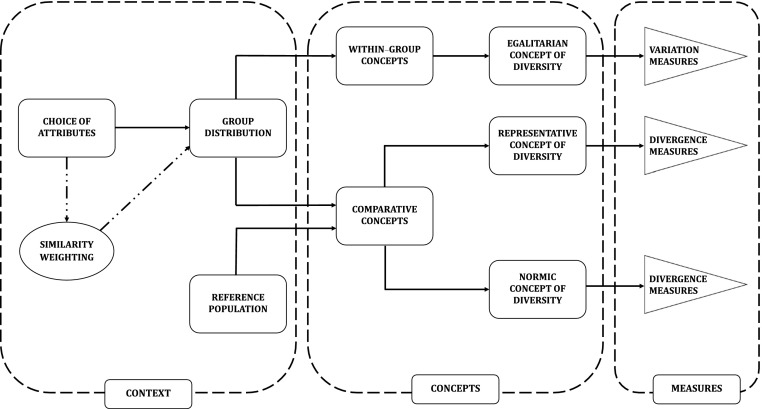
A simplified schematic depiction of the relationships among context, concepts, and measures of diversity. Arrows represent informational dependencies (e.g., a reference population is needed to apply a comparative, and hence, a representative diversity concept). Dotted lines indicate optional elements. The context suggests appropriate attributes, reference population, and similarity measures. Given a context, concepts of diversity can be divided into two general types: within-group and comparative, with the latter involving a comparison between the group distribution and properties of a reference population. Diversity measures can then be used to quantify these concepts

The three diversity concepts considered in this section also illustrate the distinction between *within group* and *comparative* diversity concepts. For within group diversity concepts, once the relevant attributes and similarity weighting (if applicable) are given, the diversity of the group in question does not vary depending on which outside reference or comparative population the assessor might have in mind. Egalitarian diversity illustrates this type of diversity concept. Harrison and Klein’s (2007) diversity as separation and disparity are also within group concepts. By contrast, representative and normic diversity concepts are comparative because they depend on a chosen reference population. For such concepts, switching reference populations can alter assessments of diversity. The attendees at Vaisakhi festivals in Vancouver might be diverse in a normic sense if the reference population is Canada as a whole, but not if it is the North American Sikh diaspora. Similarly, the faculty of a university engineering department might display gender diversity in a representative sense if the reference population is taken to be recent engineering PhDs, but not if it is university undergraduates. The relationship between within group and comparative diversity concepts is represented in Fig. 1.

Finally, the diversity concepts discussed thus far are not exhaustive. To see this, consider the possibility of similarity weighting, noted in section 2. It is natural to take a similarity weighting to be implicit within a normic diversity concept. Thus, if Christian is the non-diverse norm, then a group of Sikhs might be judged more diverse in a normic sense than a group of Jews on the grounds that Judaism is more similar to Christianity than Sikhism. Similarity weighted versions of the other concepts might also be considered. Moreover, further concepts can be generated via hybridizing egalitarian, normic, or representative diversity, as the example of deliberative mini-publics in section 5 illustrates.

## Multiple concepts of diversity in science

In this section, we show that contextually embedded versions of egalitarian, normic, and representative concepts can be found in research on diversity in science and in explanations of the effects of diversity on group performance. Moreover, we claim that distinctions among these concepts are important because separate concepts are often associated with distinct epistemic and ethical rationales for why diversity is valuable, and because failure to attend to these differences can lead to unjustified inferences.

Let us begin with an example of egalitarian diversity. In a widely cited article, Hong and Page (2004) introduce a formal model for studying the effects of diversity of perspectives and heuristics on group problem solving. Here, perspectives refer to how an agent represents the space of possible solutions that the agent could employ towards solving the problem. Heuristics refer to the way in which the agent searches through this space of possibilities. The performance of each agent is measured by the expected value of the solutions arrived at, given their perspective and heuristic. When solving a problem collectively, agents elaborate on each others’ solutions in a serial manner: an agent attempts to solve the problem, and when they are stuck at a point, another agent tries to find a further improvement. The collective stops when no other agent can find a better solution.

Exploring the consequences of this formal model computationally and analytically, Hong and Page show that, under certain conditions, a collective that is randomly drawn from a general pool of diverse problem-solvers outperforms a problem-solving team consisting of individually best-performing agents. Two points from Hong and Page’s model are worth highlighting here. The first concerns the contextual aspect of decisions about what counts as a relevant attribute. As depicted in Fig. 1, the choice of relevant attributes is contingent on the particular context of investigation; insofar as Hong and Page aim to delineate the conditions under which diversity is beneficial for tasks such as problem-solving, the relevant attributes with respect to which diversity is understood are “functional” attributes, such as perspectives and heuristics. Hong and Page distinguish these functional attributes from attributes that are relevant to what they call “identity diversity”, which include categories such as gender, race, and so on. The second point has to do with the particular concept of diversity that figures in Hong and Page’s explanation (the concept box in Fig. 1). Prima facie, the explanation seems to rely on a non-weighted egalitarian notion of diversity. This is because, given the random sampling process, the distribution of heuristics will tend to uniformity as the size of the sample gets larger. A closer look suggests a more complex situation, however. Hong and Page assess the diversity of groups by means of a measure that depends not only on whether the two heuristics differ from one another, but also on how distinct they are, where distinctness is understood in relation to the number of overlaps in the two ordered sets that represent the heuristics (Hong and Page 2004, 16,386–16,387). Accordingly, it is more apt to view the underlying concept of diversity employed in Hong and Page’s explanation as an instance of the weighted egalitarian concept.

Given these observations, we may ask a more general question about the rationale for employing a particular diversity concept in a given context. In particular, why are within-group concepts, such as a weighted egalitarian concept, suitable for “perspective and heuristics differences” explanations of the cognitive benefits of diversity? The central idea is that heterogeneity within the group can increase the breadth of its “cognitive repertoire,” which in turn can, under the right conditions, increase the quality of results (Page 2017). Kitcher (1990), for instance, suggests that scientific communities will be more productive if not every scientist works on the theory or employs the methodology with the best chance of success given the current evidence, since an alternative theory or methodology may turn out to prove more successful as new evidence emerges. Similarly, Longino (1990, 2002) argues that a diversity of value perspectives reduces the risk that unjustified assumptions will escape scrutiny, while Solomon (2001) proposes that diversity in the sense of a uniform distribution of non-empirical decision vectors within science is epistemically beneficial.

Hong and Page (2004) and Weisberg and Muldoon (2009) take such ideas further by allowing different perspectives and heuristics to combine in novel ways. In Hong and Page’s account, a group consisting of two agents can employ three different heuristics, two belonging to each of the agents and one resulting from the combination of the two individual heuristics. The core idea is the same, however: diversity in a within-group sense is beneficial because heterogeneity within the group increases the number or breadth of solutions the group is able to locate, thereby increasing the chance that a superior option will emerge.

Let us turn to normic concepts of diversity. While explicit statements of normic diversity concepts are rare, their use is fairly common. A distinctive feature of normic diversity concepts is that they render expressions such as “diverse persons” coherent. A diverse person differs from what is taken to be the non-diverse norm in a context, and hence “diverse persons” would refer to a collection of such people. This suggests a simple tactic for locating work that utilizes a normic diversity concept: enter phrases such as “diverse scientist,” “diverse student,” and so on into search engines and see what turns up.

By such means, we found articles with titles such as, “Exploring the Potential of Using Stories about Diverse Scientists and Reflective Activities to Enrich Primary Students’ Images of Scientists and Scientific Work” (Sharkawy 2012), “An ROI Comparison of Initiatives Designed to Attract Diverse Students to Technology Careers” (Dillon et al. 2016), and “Culturally and Linguistically Diverse Healthcare Students’ Experiences of Learning in a Clinical Environment: A Systematic Review of Qualitative Studies” (Mikkonen et al. 2016). A closer examination supports interpreting these articles as relying on a normic diversity concept. To support this interpretation, it is necessary that there be a reasonably clear non-diverse norm from which those characterized as “diverse” diverge. Thus, Sharkawy (2012, 309) discusses the prevalent stereotype of scientists as “old, white eccentric males of extraordinary intelligence,” which “exclude many groups of people including, for example, females, wheelchair-bound students or those with other physical challenges, students from non-Caucasian backgrounds and students who do not consider themselves of superior intelligence.” The stereotype is an expression of the non-diverse norm, and the diverse scientists and students are those it excludes. In a similar vein, Dillon et al. (2016, 105) motivate their work in response to calls to recruit “underrepresented populations, specifically minorities and women” into STEM fields. In this example, minorities and women are diverse students relative to the non-diverse norm of white males. However, the non-diverse norm is not always a variation on “white male.” In review by Mikkonen et al. (2016) of work on “cultural and linguistic” diversity among nursing students who study abroad, the non-diverse norm is linked to the country in which the students study. For example, nursing students from the UK would be culturally and linguistically diverse in Sweden, and vice versa (Mikkonen et al. 2016, 179). The choice of non-diverse norm, therefore, can vary according to context.

In addition, some explanations of positive cognitive effects of diversity appear to use a normic diversity concept. The first of these that we consider is standpoint epistemology. Though several variants of standpoint epistemology exist, a common theme is that members of marginalized groups are more likely than their dominant counterparts to develop standpoints that accurately depict the workings of unjust power structures (Collins 2004; Crasnow 2008; Harding 1998, 2004, 2015; Intemann 2010a, 2010b; Rolin 2006, 2016; Wylie 2003, 2011). To illustrate, consider an expert panel convened to address issues of sexual harassment in a white male dominated field. Standpoint epistemology suggests that a group of women—especially one including minority women—would be more likely than a group of white men to produce effective proposals on this topic. Standpoints are not automatic by-products of social locations, but the result of a critical interaction among a group of people driven by a shared goal of opposing oppression (Wylie 2003).

The thesis that members of socially dominant groups often tacitly accept viewpoints or social practices that tend to obstruct knowledge suggests an instantiation of normic diversity in which the dominant group is the non-diverse norm, while the diverse people are those who tend to be marginalized or oppressed. Harding endorses such a view of diversity when she writes:

What is the diversity on which I focus here? … one central concern is to include in scientific decision making the groups that heretofore have been excluded from participating in decisions about research that has effects on their lives. (Harding 2015, xi)

Here those who have traditionally dominated a scientific field would be the non-diverse norm, and those who have been excluded would be the diverse people. Harding contrasts diversity in this sense with what she terms “mere diversity,” which would require including a wide range of political perspectives, including oppressive ones such as white supremacism (Harding 2015, 35). Harding does not define “mere diversity,” but it could be interpreted to refer to the number of perspectives present in the group or the degree of divergence between them. It could also mean egalitarian diversity, which can be increased by extending the number attribute categories present, as noted in section 2.^8^

Normic diversity concepts also appear in some empirical work on diversity. Consider research suggesting that certain interaction styles—for instance, those exhibiting greater social sensitivity and egalitarianism in speaking turns—tend to promote cognitive performance of groups and tend to be associated with certain social identities, such as female gender (Bear and Wooley 2011; Engel et al. 2014; Woolley et al. 2010). Such explanations resemble standpoint epistemology in linking social dominance to an epistemically harmful trait (an insensitive or authoritarian interaction style in this case), which can thereby generate an epistemic advantage for non-dominant or oppressed individuals. Consequently, these explanations suggest a normic conception of diversity for reasons similar to those discussed in connection with standpoint epistemology.

Since egalitarian and normic diversity concepts are often associated with distinct explanations of how diversity enhances group performance, failure to distinguish between the two can result in problematic inferences. Consider a concrete example. In a literature review of diversity research in occupational demography, Joshi and Roh (2009, 615) report that increased gender diversity is more likely to generate negative effects, such as social discord, in predominantly male settings than in more gender-balanced groups. They also find a similar effect for ethnicity: increasing ethnic diversity is more likely to have negative effects when the group is predominantly white (Joshi and Roh 2009, 615). They take this to support the hypothesis that “the negative effects of gender and race/ethnicity diversity [is] weaker in more gender-balanced and ethnically balanced settings, respectively” (Joshi and Roh 2009, 615). Joshi and Roh interpret “diversity” in an egalitarian sense.^9^ Thus, their hypothesis predicts that introducing a small number of men into an overwhelmingly female group would be more likely to produce negative effects than similarly increasing the number of males in a group that is more gender-balanced. But the data Joshi and Roh (2009) review are insufficient to support this prediction, as it is unclear whether the observed effect is due to homogeneity per se or to a strong numerical majority of a socially dominant group. Since homogeneity is the opposite of egalitarian diversity, while normic concepts often link non-diversity to social privilege or dominance, this example illustrates the importance of considering both egalitarian and normic diversity concepts, and the distinct types of explanation they tend to be associated with.

Let us turn, then, to representative diversity. In a literature review of conflicting empirical results regarding the effects of diversity on group performance, Smith-Doerr and colleagues write, “Representational diversity, where organizations have workforces that match the pool of degree recipients in relevant fields, is a necessary but not sufficient condition for diversity to yield benefits” (Smith-Doerr et al. 2017, 140). They suggest that “full integration,” which involves interactions and communication on socially equal terms, is also necessary for demographic diversity to yield positive impacts (ibid.). Note that Smith-Doerr and colleagues’ definition of representational diversity specifies a reference population, namely, recent graduates in the field. By contrast, representative diversity as we understand it leaves the choice of reference population open.

Representativeness is also one way to understand what it is to report a diverse range of viewpoints in journalism (Baden and Springer 2017; Jacklin 1978). Thus, according to Jacklin, “There is representative diversity when the political diversity in communications is *representative* of the political diversity in the total society” (Jacklin 1978, 87; italics in original). Note again the specification of a reference population in this definition (“the total society”), which contrasts with the more general representative diversity concept articulated in section 3. Representative diversity can be understood to apply to the people who express the views or to the time devoted to the views expressed. Democratic legitimacy is an obvious rationale for a representative interpretation of diversity (Jacklin 1978). However, there can also be epistemic motives for interpreting diversity in a representative sense. For example, journalists are sometimes criticized for giving equal attention to mainstream climate scientists and climate change sceptics, rather than conveying a representative picture of scientific views (Boykoff and Boykoff 2007; Boykoff 2013). Such claims suggest that representative diversity may be epistemically justified when a skewed distribution of viewpoints in a reference population is driven by evidence. This example also illustrates the significance of which reference population is chosen. Representative diversity would suggest very different distributions of views regarding climate change depending on whether the reference population is taken to be climate scientists or the general public.

In this section, we have shown that egalitarian, normic, and representative diversity concepts can all be found in research on the effects of diversity. We claim that this has significant implications for philosophical work on the question of whether diversity promotes better science. In this context, failure to attend to distinct diversity concepts can result in unjustified inferences from empirical data, as can happen when a comparison group is non-diverse in both an egalitarian and normic sense. Moreover, distinguishing among diversity concepts helps to render visible value decisions that might otherwise remain hidden, such as those relating to choice of the reference population or non-diverse norm. Finally, what counts as diverse can vary drastically given egalitarian, representative, and normic diversity concepts. When the distribution of relevant attributes in a reference population is skewed in favor of a dominant group, diversity in a representative sense can look like abject tokenism from an egalitarian or normic perspective. And a normic conception is compatible with rejecting the egalitarian insistence that diversity entails a uniform distribution. The next section considers an example that further elaborates these ideas.

## Diversity in deliberative mini-publics

Deliberative mini-publics are exercises in which members of the general public are recruited to learn about a policy issue, discuss the issue among themselves, and then provide input to the decision-making process. A number of models for deliberative mini-publics have been developed and used since the 1990s, including citizens’ juries, consensus conferences, and deliberative polling (Goodin and Dryzek 2006). Deliberative mini-publics have also been used in connection with policy decisions related to science and technology, such as human tissue biobanking (Burgess 2014; Longstaff and Burgess 2010; O’Doherty and Burgess 2013; O’Doherty and Hawkins 2010). In this section, we illustrate how deliberative mini-publics raise coupled ethical-epistemic issues linked to the choice of diversity concept, making clarity and transparency about these concepts useful.

References to diversity often arise in connection with discussions of deliberative mini-publics (Burgess 2014; Longstaff and Burgess 2010; Goodin and Dryzek 2006). Given the link between representativeness and democratic legitimacy, it is natural to interpret “diversity” in a representative sense in this context. However, while there is something right about this idea, the matter is more complex than it might initially appear. For example, Goodin and Dryzek write:

We will be focusing on mini-publics with some claim to representativeness of the public at large. … By “some claim,” we do not mean statistical representativeness—which only one design, the deliberative poll, explicitly asserts. … All “some claim to representativeness” need mean is that the diversity of social characteristics and plurality of initial points of view in the larger society are substantially present in the deliberating mini-public. Social characteristics and viewpoints need not be present in the same proportions as in the larger population. (Goodin and Dryzek 2006, 221)

This passage has several implications for how diversity should be understood in connection with deliberative mini-publics. First, it specifies something about the relevant attributes, which are to include both demographic groups and perspectives present in the broader society. Second, it endorses what one might call an attenuated representative diversity concept. According to this concept, the relevant attribute categories present in the broader reference population (e.g., ethnic groups, points of view) should also be present in the deliberative mini-public, but not necessarily in the same proportions. For example, it may be important for Australian Aboriginal people to be included in a deliberative mini-public held in Australia, but possibly in a greater proportion than present in the Australian population at large.

One way to understand such an approach is as a hybrid of representative and egalitarian or normic diversity concepts. The attribute categories that matter to diversity are determined by those that are present in the reference population, while their distribution in the deliberative mini-public may be guided by an egalitarian or normic concept, or some combination of the two. Consider these three hybrid concepts: (a) a representative/egalitarian hybrid in which only the attribute categories present in the reference population matter, but these categories should be distributed in equal proportions in the deliberative mini-public; (b) a representative/normic hybrid in which the distribution of attribute categories is similar to the reference population except that proportions of certain historically marginalized or discriminated against groups are higher in the mini-public, and (c) a representative/egalitarian/normic hybrid, which is similar to (a), but with some marginalized or discriminated against groups present higher proportions in the mini-public than members of dominant groups. Moreover, a similarity weighting could be combined with any of these three hybrid concepts.

Decisions about which diversity concept to adopt raise coupled ethical-epistemic issues. While democratic legitimacy is a motivation for a representative diversity concept, strictly adhering to representative proportions in a deliberative mini-public can be problematic when the distribution in the reference population is heavily skewed in favor of one or a few attribute categories. In such cases, minority perspectives are likely to be drowned out in a strictly representative deliberative mini-public, thereby incurring the ethical and epistemic risks this entails. Such considerations might be taken to support the representative/egalitarian hybrid concept (a). However, there may be circumstances in which the inclusion of certain historically historically discriminated against or marginalized groups is deemed especially important. This may be because members of these groups are likely to possess knowledge or insights that are typically absent among members of more socially dominant groups, or because members of a non-dominant social group might be more likely to use social interaction or communication styles that facilitate effective deliberation. Such considerations could support the choice of hybrid concept (b). On the other hand, a representative/normic hybrid might give inadequate voice to perspectives of who have been less subject to discrimination, which might suggest a representative/egalitarian/normic hybrid (c).

Consider the above in connection with deliberative mini-publics convened to address ethical issues arising from the creation of the BC BioLibrary in the Canadian province of British Columbia in 2007, which conserves samples of human tissue for health research purposes (Burgess 2014; Longstaff and Burgess 2010; O’Doherty and Burgess 2013; O’Doherty and Hawkins 2010). Published work discussing these events frequently emphasizes representativeness and diversity in connection with recruitment. For example, O’Doherty and Hawkins write:

The aim of recruitment was to achieve a sample that represented the diversity of values, life experiences, and discursive styles of the citizens of British Columbia... A further aim was to address perceived democratic deficits, by giving voice to individuals and groups that would otherwise not be heard. (O’Doherty and Hawkins 2010, 202; cf. Longstaff and Burgess 2010, 216; O’Doherty and Burgess 2013, 78)

Longstaff and Burgess (2010, 218) also emphasize including voices that tend to be marginalized. O’Doherty and Hawkins describe the resulting recruitment process as follows:

Thresholds were used to achieve approximate proportional representation relative to official Canadian census statistics, with the exception of two groups (First Nations and individuals with genetic or chronic disabilities). These two groups were over represented relative to the general population (minimum of two participants for each category) to ensure that their voices would be present on a topic potentially able to affect them in a disproportionate manner. (O’Doherty and Hawkins 2010, 202)

This procedure is, in effect, an application of a hybrid representative/normic diversity concept.

Carefully articulating and distinguishing egalitarian, normic, and representative diversity concepts is helpful in this context. First, it can contribute to greater clarity about what is meant by “diversity,” a term which is not defined in any of the papers on deliberative mini-publics and biobanks that we cite. Conceptual unclarity about diversity renders phrases such as “maximizing the diversity of participants” (O’Doherty and Burgess 2013, 60) or “participation of diverse publics” (Burgess 2014, 50) difficult to interpret, and makes it difficult to assess claims about the diversity of participants (cf. Longstaff and Burgess 2010, 218–219; O’Doherty and Burgess 2013, 60). Second, explicit awareness of multiple diversity concepts and the ethical and epistemic rationales that may be provided for them is useful for decisions about which concept to adopt in a context. A fuller suite of options can be considered, and reasons for choosing one over another can be expressed.

## Conclusions

This article has distinguished egalitarian, representative, and normic diversity concepts and explored their interconnections. The differences between these concepts matter, because they are often linked to separate explanations of why diversity is desirable from an ethical or epistemic perspective, and because variants of egalitarian, representative, and normic concepts are embedded in specific contexts related to diversity in science. Consequently, inattention to differences among these concepts can generate ambiguity about what “diversity” means or problematic inferences when an explanation associated with an alternative diversity concept is not considered. Moreover, since these three concepts have not previously been jointly considered, the possibility of generating further hybrid diversity concepts from them has not been examined either. The example of deliberative mini-publics suggests that hybrid diversity concepts (e.g., which combine representative and normic elements) may be useful. Such hybridization vastly expands the options for diversity concepts, further accentuating the possibility of ambiguity and underdetermination. A closer look at concepts, therefore, is imperative for research on diversity.

## Footnotes

1 As we discuss below, context can also suggest other considerations relevant to diversity, including a similarity weighting or a comparison population.

2 These categories might not be regarded as mutually exclusive, e.g., because one could identify as both Christian and Muslim. If one wished to consider such possibilities, the attribute categories could be taken to be all combinations of the affiliations. But if the intersections are very sparsely populated, single affiliations might be treated as mutually exclusive.

3 The two concepts being how flat the distribution is and how many attribute categories are present.

4 Blau’s index is the following: $D=1-{\sum}_i^n{p}_i^2,$where *p*_*i*_ is the proportion of members of the group who are *a*_*i*_ (cf. McDonald and Dimmick 2003, 61; Teachman 1980, 343).

5 Although answer 3 does differ from the others in focusing on sects within Sikhism.

6 In examples in which multiple attributes are considered, the non-diverse norm would more likely be represented by a cluster of categories of different attributes, such as cisgender heterosexual able Christian white male.

7 Page also sometimes uses a concept of diversity similar to complementarity, according to which a person p is diverse with respect to a group G if p’s cognitive repertoire does not overlap with those of the members of G (Page 2017, 96). If cognitive repertoire is the relevant attribute, then in such a case, G ∪ {p} is more diverse than G according to Blau’s index (i.e., more diverse in an egalitarian sense).

8 Relatedly, Intemann states that feminist empiricism and standpoint epistemology rely on different “kinds of diversity”: feminist empiricists focus on values and interests, while standpoint epistemology emphasizes social position (Intemann 2010a, 790). Prima facie, this is a distinction between types of attributes, rather than among diversity concepts as understood here. However, Intemann’s text is consistent with attributing an egalitarian concept to feminist empiricism and a normic concept to standpoint epistemology.

9 They endorse a within group concept of diversity similar to Teachman’s “population diversity” (Joshi and Roh 2009, 600). In addition, they use Blau’s index and Teachman’s (1980) entropy measure to assess diversity for qualitative attributes (Joshi and Roh 2009, 612). Blau’s index is a measure of egalitarian diversity for single qualitative attributes as noted in section 2, while Teachman’s entropy is an extension of Blau’s index to examples involving more than one attribute.
